# Mitigating stereotypes and bias in professional identity formation for those with marginalized identities

**DOI:** 10.1016/j.rcsop.2024.100434

**Published:** 2024-03-18

**Authors:** Natalie Rosario, Joshua Wollen

**Affiliations:** Pharmacy Practice and Translational Research, University of Houston College of Pharmacy, Health 2 4349 Martin Luther King Boulevard, Houston, TX 77204-5039, USA

**Keywords:** Professionalism, Identity formation, Underrepresented in pharmacy, Stereotype, DEIA

## Abstract

Pharmacists have many identities within the profession from medication experts, clinicians, educators, mentors, patient advocates, and more. It can be especially challenging for racially and ethnically minoritized persons (REMPs) to form a professional identity when they are surrounded by stereotypes and biases which are pervasive in the community, academia, and pharmacy practice settings. As pharmacist educators, preceptors, and mentors, it is important to create safer spaces that decrease stereotyping and biases for students so they may envision themselves thinking, acting, and feeling like a pharmacist. Here, literature on professional identity formation in underrepresented groups in the United States is reviewed to continue the conversation of creating safer spaces for underrepresented students as they develop their professional identity.

## Highlights

• Pharmacy academia has increased focus on professionalism and professional identity formation (PIF).
• Learners from underrepresented backgrounds may have a different path to PIF based on prior experiences in or around healthcare settings.
• Underrepresented learners may be subjected to stereotypes and bias from academic institutions when formulating their PIF.
• Educators must be mindful and conscious of their own biases when introducing PIF to diverse learners.

## 1 Introduction

Pharmacists may take on many identities in their professional careers such as medication expert, clinician, scientist, educator, researcher, manager, businessperson, mentor, advocate, and carer.^1^^,^^2^ Student pharmacists and new graduates take on roles such as learner, leader, speaker, health promoter, teammate, intern, information master, and patient care advocate.^2^ Student pharmacists in the United States (US) from underrepresented or marginalized identities may struggle to see themselves with these labels from lack of exposure to professionals who look like them. A qualitative study of 29 physician role models by Wright and colleagues (2003) describes that from the role model perspective, “learners prefer role models similar to them, role modeling is easier when the learner resembles the teacher, and minority physicians may be better role models for minority learners.”^3^ The American Association of Colleges of Pharmacy and the Association of American Medical Colleges define underrepresented as “racial and ethnic groups that are underrepresented in the profession in comparison to their numbers in the general population.”^4^^,^^5^ Underrepresented in pharmacy or medicine learners include Black or African American, Hispanic or Latine, and Native American or American Indian populations.^4^^,^^5^ This commentary will refer to these groups as racially and ethnically minoritized persons (REMPs)^6^ in this manuscript. In 2017–2018, 9.1% of student pharmacists identified as Black or African American, 6.5% as Hispanic or Latine, 0.2% as Native Hawaiian/Other Pacific Islander, and 0.4% as American Indian/Alaska Native.^7^ The discrepancy in makeup of REMP cohorts in healthcare professional education can impact how REMPs develop their professional identity.

First, it is critical to review professionalism as this is distinct from professional identity formation (PIF). Professionalism includes core values and norms, such as compassion, integrity, honesty, respect, accountability, teamwork, lifelong learning, and effective communication.^8^^,^^9^ The American Society of Health-System Pharmacists and Hammer and colleagues (2006) define pharmacy professionalism characteristics of “competence, inclusivity, ethics, accountability, integrity, trust, responsibility, initiative, maturity, and interpersonal communication skills.”^10^^,^^11^ Patients value pharmacists who are “friendly, helpful, trustworthy, professional, competent, caring, knowledgeable, responsive, and approachable.”^12^ Professionalism is the outward demonstration of these attitudes and behaviors. Professionalism is not dictated by volume of speech or physical appearance, but rather on interpersonal skills. These values and norms impact how students perceive the profession of pharmacy and how they will see themselves as future pharmacists. The authors support that professionalism pertains to attitudes and behaviors and should not be focused on symbolic manifestations related to physical appearance such as hair or tattoos whether modifiable or not.^13^^,^^14^ Physical attributes do not determine a students' professionalism or competence. Personal biases that uphold unfounded Eurocentric preferences related to physical appearance should be challenged to create inclusive spaces for those from REMP identities.

In contrast, PIF suggests that students “think, act, and feel” like a pharmacist^15^ which is the internalization of being a pharmacist. The embodiment of a pharmacist role is influenced by the person's socialization such as personal experiences, friends and family, social isolation, role models, mentors, interactions with the healthcare system, and social media.^15^ Socialization begins in childhood and influences a persons values, behaviors, and perspectives. This socialization plays an integral role in how learners approach professionalism and professional identity formation.^15^^,^^16^ Thus, PIF may look different to individuals based on their background. REMPs may have had negative experiences with the healthcare system, potential for social isolation due to systemic racism, and may not have had access to mentors and role models who look like them in their pursuit of a career in healthcare.^3^ Additionally, REMPs may have a history of being subjected to stereotypes, bias, and generalizations.^17^ REMPs may then have a harder time assimilating into the PIF ideology since it may be challenging to “think, act, and feel” like a pharmacist in academic and healthcare systems that have historically marginalized their identities. It is difficult to develop a professional identity if there are conflicts between the personal identity and the professional identity the student is working to assume.^18^ For example, differential attainment or the attainment gap has been evaluated in pharmacy and medical education.^19^^,^^20^ This commentary aims to bring awareness of how stereotyping and bias related to underrepresented groups in PIF publications can cause harm in REMPs in order to avoid stereotypes or harmful generalizations to minimize future harm to learners.

## 2 The emergence of PIF in healthcare professional education

Due to the recent implementation of PIF in health professions education and the interest it has garnered, educators have been exploring PIF in many capacities. One emerging trend is examining the connection between PIF and REMPs. In doing so, racial or ethnic harm may occur from the manner in which these works are produced. While PIF itself may raise conceptual concerns, this work describes harm that comes from *how* these discussions take place.^21^

There have been calls to incorporate PIF into pharmacy education over the past decade including various review articles and task force or committee reports from the American Association of Colleges of Pharmacy.^22^, ^23^, ^24^, 25., 26, 27 Since then, these calls have been answered by scholarship related to PIF's integration into Colleges of Pharmacy in the US.^28^ Esposo and colleagues (2023) studied pharmacy skills lab activity learning objectives from all lab courses at 6 US Colleges of Pharmacy finding that “healthcare provider” was the most prevalent professional identity identified in laboratory curricula taught through skills-based learning activities despite most activities in the sample were categorized as the “prepare / dispense / provide medications” domain.^29^ While PIF occurs during skills-based learning, learning objectives may not support the intended professional identity targeted by the activity. Zaudke and colleagues (2016) developed an integrated interprofessional education (IPE) experience and found that professional identity scores from the Readiness for Interprofessional Learning Scale improved for the students in the integrated IPE group.^30^ These findings suggest that integrated IPEs contribute to the development of professional identity. PIF is also being incorporated outside the Doctor of Pharmacy curriculum at some institutions. Mekonnen and colleagues (2023) described a PIF-related co-curricular program and analyzed the student's reflections for Accreditation Council for Pharmacy Education Standards 3 and 4 which are described as “Approach to Practice and Care and Personal and Professional Development,” respectively.^31^^,^^32^ Arnoldi and colleagues (2022) mixed methods study analyzed reflection essays from first and second professional year students. The students felt the co-curricular experiences had the greatest impact on their PIF.^33^ These examples also demonstrate how PIF is incorporated into Colleges of Pharmacy *both* passively (i.e., PIF has always unintentionally taken place) and actively (i.e., explicit endeavors to develop professional identity).

When discussing PIF in the context of REMPs, authors may unintentionally stereotype when using REMPs as props to describe scenarios. Recommendations from these works perpetuate harm in an effort to integrate REMPs within a profession that historically has excluded them using a framework that requires them to assimilate.^21^^,^^34^^,^^35^ These issues extend beyond PIF-related texts but is particularly concerning given the increase in PIF literature related to REMPs. Addressing this now creates an opportunity to mitigate further harm during the ongoing focus on PIF in health professions.

## 3 Avoiding stereotypes in PIF: student generalizations & institutional accountability

Much PIF research has not specifically studied REMPs. Table 1 synthesizes the available literature of PIF in underrepresented groups including pharmacy, medicine, and physician assistants.^21^^,^^36^, ^37^, ^38^, ^39^, ^40^, ^41^, ^42^ When studying PIF in REMP groups, it is critical to avoid stereotypes. Stereotyping learners while asking them to develop a professional identity can contribute to stereotype threat, where minoritized learners are subjected to high cognitive load and decreased academic focus as a means to avoid enacting a stereotype behavior.^17^^,^^43^

**Table 1:** – Professional identity formation in underrepresented health professionals.

Author, year	Profession	Intervention and Demographics (%)	Key Themes
Gunaseelan, 2023^18^	Pharmacy students in Texas	38 student PIF reflections were evaluated. 35 (92%) Hispanic 3 (8%) Black / African American	− Students had defining experiences which led to realizations of the importance of pharmacists who reflect their patient populations in culture and language. − Students had internal motivating factors to return to their communities (ie: small town, underserved). − Students expressed aspirations to work as pharmacists in underserved communities to increase equity and accessibility, provide extensive counseling on diabetes and heart disease, and to communicate with patients in Spanish. Students expressed needing to overcome stereotypes based on their physical appearance, familial educational background, and English-language proficiency (ie: having an accent).
Malhotra, 2023^29^	Undergraduate	Black students	Three frameworks are reviewed related to how PIF in pharmacy can be inclusive to Black students: − **Tinto's framework:** student feelings of belonging can be cultivated through socialization (ie: campus activities, cultural support, mentorship with faculty, and inclusive pedagogy). − **Arroyo and Gasman's HBCU-institutional framework**: HBCU's have a history of creating accessible and economically reasonable entry points for Black students. Additionally, HBCU's offer more supportive environments which allow facilitate academic success, instills values, reinforces community service, servant leadership, effective communication, and professional advocacy. − **Bank's multicultural education theory:** curriculum that includes lived experiences related to students' identities is valuable given marginalized students may have overcome additional obstacles in their journey to reach their professional goals.
Rockich-Winston, 2023^30^	Pharmacy students in Georgia and Illinois	15 students were interviewed. **Gender:** − 12 female (80%) **Race / ethnicity:** − 11 (73.3%) Black/ African American − 2 (13.3%) Hispanic/Latine − 2 (13.3%) more than 1 race / ethnicity	**Disadvantages based on identities:** − Feelings of inadequacy, having to try harder than peers, and having less privilege based on race / ethnicity compared to peers − Always putting best foot forward due to others counting her out − Pressure of perfectionism to ensure work and reputation is not questioned. It is more acceptable for peers who do not look like her to make a mistake − Correcting misinformation on affirmative action − Expending energy disproving stereotypes or accusations of cheating − Balancing authenticity without exhibiting stereotypes − Concerns that in patient care settings they may experience overt racism from patients − Lack of representation or support in student organizations for students of color, which led to the creation of a new student organization − Internal and external conflict with biracial identity − Lack of representation in the profession, within pharmacy school faculty, and in patient cases **Impact of identities in PIF** − Desire to work in underserved communities and feeling a need to advocate and protect underserved communities − Promote inclusion (ie: counseling a patient in Spanish) − Having family members in healthcare as an inspiration to pursue pharmacy as a career − Desire to mentor others from similar identities − Having faculty member from similar background can impact matriculation in the pharmacy program
Thomas, 2023^31^	Pharmacy students	Underrepresented in pharmacy	− Students from marginalized identities may feel pressured to conform to profession norms which may not be their own cultural norms. − Identity dissonance may appear as students balance personal identity and professional identity which may be incongruent. − Strategies to strengthen PIF in underrepresented groups may include mentoring, socialization to the profession, providing learners opportunities for self-reflection and feedback, and being cognizant and respectful of cultural traditions.
Wyatt, 2020^32^	Medical students in Georgia	14 African American / Black medical students. **Gender:** − 2 female (14.3%) − 12 (85.7%) male	− Black / African American students engage in identity negotiation of self-perception being Black and joining the profession of being a physician − URM students experience “minority tax” when developing their professional identity − URM students experience “double consciousness” where they process how others from a dominant group view them − Black students mentored other Black students who were interested in pursuing medicine − Stereotypes about racial identity are pervasive and Black students have to work twice as hard to dispel stereotypes as assumptions are made about the student before having any interactions − Black students adjusted their physical appearance (hair, make up, clothes) and normative behaviors (speech, inflection, body movements) − Black students experience a large mental burden of adjusting social cues which can be isolating and exhausting − Integrating leadership within the Black community contributes to professional identity (mentoring, tutoring, service) − Black students often feel more comfortable receiving mentorship from physicians who look like them, but may seek out mentorship from majority group members − When schools do not comment on sociohistorical moments (ie: police brutality) it can lead to feelings of being invisible
Wyatt, 2021^33^	Medical students and physicians	41 **physician** professionals interviewed **Training status:** − 14 (34.1%) students − 10 (24.4%) resident − 17 (41.5%) clinician **Gender:** − 31 (75.6%) female **Race / ethnicity:** 41 (100%) Black / African American	− Due to medicine's history of exclusion, Black medical students are conditioned to watch their backs and keep their eyes open as medicine may not be safe for them − Black students or physicians are mistaken for custodial staff, nurses, or other staff members − Attending physicians may also feel disconnected from being both Black and a physician − Black staff members look out for the Black physicians giving hints to keep their eyes open − Black physicians and students spend much time intentionally mentoring and helping other Black and brown trainees with the hopes of creating a sense of belonging within the profession − Racial uplift is prevalent in Black medical students and physicians core values to help others to achieve their goals
Wooten, 2023^34^	Physician Assistant (PA) students and PAs	45 **PA** professionals interviewed. **Training status:** − 26 (60.5%) students − 17 (37.8%) clinician **Gender:** − 32 (71.1%) female **Race / ethnicity:** − 23 (51.1%) Black / African American − 12 (26.7%) Latine − 4 (8.9%) Indigenous / Native − 6 (13.3%) mixed race / ethnicity	− Racial concordance between provider and patient increases trust given similar cultural understanding − Language concordance between provider and patient leads to positive patient outcomes − Understanding cultural taboos (ie: surgery in Indigenous / Native communities) can help facilitate conversations around sensitive topics − Native / Indigenous PAs experience microaggressions when other providers make generalizations about the community without cultural understanding − Students are stereotyped by preceptors (ie: comments about hair styles, names the preceptor cannot pronounce, or comments about work ethic based on ethnicity) which makes the learning and clinical environment unsafe − Minoritized students may feel tokenized and heightened pressure to perform well for the sake of future minoritized students at that site
Wyatt, 2022^35^	PA students, practicing PAs, medical students, and physicians	45 **PA** professionals interviewed **Training status:** − 26 (60.5%) students − 17 (37.8%) clinician **Gender:** − 32 (71.1%) female **Race / ethnicity:** − 23 (51.1%) Black / African American − 12 (26.7%) Latine − 4 (8.9%) Indigenous / Native − 6 (13.3%) mixed race / ethnicity 41 **physician** professionals interviewed **Training status:** − 14 (34.1%) students − 10 (24.4%) resident − 17 (41.5%) clinician **Gender:** − 31 (75.6%) female **Race / ethnicity:** − 41 (100%) Black / African American	**Similar themes between underrepresented PAs and physicians** − Racial identity is important to clinical care and can help minoritized patients feel more comfortable to have a provider similar to them − URM groups experience microaggressions / discrimination from peers and patients (ie: having an accent, being confused for custodial staff, being labeled unprofessional for asking questions, being overlooked or ignored) − Feeling the need to be twice as good compared to non-minoritized peers leading to increased stress to feel worthy to be in the profession − Strategies of PIF include racial uplift via mentoring − Advocating for high quality patient care for minoritized patients − Racial identity may provide a connection between patients who look like them where the patient interview feels more like a conversation as described by a Black physician and Indigenous / Native PA student **PAs** − PA professionals felt they could bring their full selves (race, gender, sexuality, religion, social history) in educational and clinical practice settings whereas physicians felt split between personal and professional identities **Physicians** − Feeling connected to patients by having daker skin and speaking their language (ie: Spanish) − Hospital settings can be especially problematic for micro-emotions where team members may create unsafe spaces for Black students by their facial expressions towards or about Black students − Feeling a sense of responsibility for being a Black man and how others may perceive those identities despite being a physician − The “culture of medicine” and standardization may contribute to social identity threat

Some PIF examples in underrepresented groups lack sensitivity to cultural identities or could be interpreted as putting the responsibility of “shortcomings” onto the minoritized student versus the system that the student is subjected to. One example includes:

“Mentors should consider the limitation on assuming leadership positions because of stereotype threat by the student and lack of role models who may look like them. For example, a first-generation minoritized student (e.g., Hispanic, Latin/a, etc) student who is interested in a specialty area of pharmacy practice… and has no immediate/extended family in healthcare may need additional nurturing because they don't see themselves in such a role.”^21^

In this example, a marginalized identity, Hispanic, Latin/a, did not need to be explicitly called out as this could be said about any student from a marginalized background. Calling out two identities of first generation and Hispanic, Latin/a, can perpetuate the stereotype that all Hispanic students are first generation and lack support when navigating higher education. The term nurturing in this context can also be interpreted as paternalistic and infantilizing the student. Additionally, this example implies that the Hispanic, Latin/a student is lesser-than based on lack of a familial connection to the healthcare system which is an unfounded claim. The authors recommend cultural sensitivity and conscientiousness when selecting examples that identify a specific minoritized identity and to reflect on whether the example scenario or suggested solution perpetuates harmful stereotypes.

Another example that could be reframed is the claim that “Ethnic/racial minoritized students spend more energy on assimilation rather than career advancement which can lead to halting their post-graduate training because of stereotype threat or feeling they lack safety in their training environment. For example, a student who is not a US citizen but is interested in post-graduate training may be discouraged from applying to a program on the basis of their immigrant visa status or worry that letter writers may not highlight their capacity for success because differences are seen as a deficit rather than an asset.”^21^ This example unintentionally places the blame on the student for spending energy on assimilation over career advancement. It can be interpreted as discrediting ethnically and racially minoritized students for not pursuing the post-graduate training versus calling out health systems, programs, and policies that uphold systemic racism that exclude applicants or make pursuing post-graduate training undesirable from a psychological safety lens. This systemic racism may prevent REMPs from pursuing post-graduate training. For the example of a student without US citizenship, 68% of residency programs do not provide information that is critical for non-US citizens, international, or refugee applicants to know if their application will be considered for review based on their citizenship status.^44^ Additionally, 21% of residency programs explicitly require US citizenship status or “greencard” equivalent.^44^ These are additional barriers that students must overcome not from lack of drive, motivation, or desire, but rather due to systemic barriers impacting their eligibility or ability to pursue postgraduate training.

When calling out personality characteristics, for example, “a minoritized student could be labeled ‘too loud’ or ‘too aggressive’ rather than being seen as assertive…” there are two sides of the continuum. Some racially or ethnically minoritized students may also be labeled “too shy, too quiet, too reserved, or too timid.”^21^ Letters of recommendation have been noted to have some gender bias related to terms such as “desire” and “solidarity / reserved,” but this has not been evaluated based on students race or ethnicity.^45^ These personality characteristics are based on Eurocentric norms as to what is acceptable in the workplace, but students may be protecting their peace and acting in a manner to not be disruptive for fear of repercussions.^17^^,^^46^, ^47^, ^48^ These unspoken rules may influence students' behaviors which leads them to censor their authentic selves.

## 4 The impact of cross-racial mentoring and interactions on REMPs

Some PIF authors discuss the importance of cross-racial interactions in PIF for reasons such as providing PIF growth for faculty as individuals, diversity of thought and mentorship, and to avoid putting this labor on marginalized faculty.^21^ While these are honorable goals, the authors argue that creating spaces where learners can be their authentic selves while learning how to be a pharmacist is essential and supersedes the rationale provided for forced cross-racial mentoring especially for underrepresented students who are African-American, Latine, Indigenous, or Pacific Islander.^49^ Experts in antiracism training and development suggest learning from each other in planned and facilitated ways where the learning is done among affinity groups. This facilitates growth alongside those that are racially or ethnically similar to them so that the groups can each develop and do their own group's labor.^50^^,^^51^ Additionally, the affinity group approach provides an opportunity to create welcoming and safe Black and brown spaces for underrepresented students to develop rather than forcing them into another space that requires temporary social assimilation. Mallory (2023) describes this temporary social assimilation as the “burden of Blackness,” the requirement to “tone it down” and not express yourself in your true ethnic identity when members of or adjacent to the dominant culture are around because of extreme social, political, occupational, and safety implications.^52^

Non-marginalized faculty should seek their own growth and development in providing inclusive racially and ethnically safe mentoring as this is not the responsibility of marginalized students to provide cross-racial mentoring practice to peers, faculty, staff, or preceptors. Prioritizing cross-racial relationships for PIF development may subject the marginalized student to additional microaggressions, stereotypes, implicit insinuations of cultural inferiority, behavior policing, and misalignment of priorities or goals from the person with socioracial power. Protective factors in PIF development in medical educators are strong mentorship, communities of practice, affinity groups, and a sense of belonging.^53^^,^^54^ These can also be applied to PIF in underrepresented groups to provide a support system and safe environment for navigating a professional identity.^17^

When faculty provide mentoring to marginalized students, they can assist students in fighting feelings of imposter phenomenon by redefining success beyond grades, attributing success to hard work over luck, and reinforcing that setbacks may occur along the journey.^55^^,^^56^ When cross-racial mentoring experiences are already in place or occur organically, deliberate and careful attention should be given to how marginalized students are mentored to facilitate student success. Additionally, it is imperative to pair constructive or corrective feedback on assignments or tasks with encouraging feedback to develop skills and build confidence in abilities.^57^ Though mentoring takes all of us, forcing integrated interactions for development is disproportionately troublesome for underrepresented students who will likely face the brunt of microaggressions from persons of power in a cross-racial mentoring interaction.

## 5 Conclusion

This commentary brings attention to the growing PIF literature in REMPs while offering another perspective. This work is important and needs further development. The goal of this commentary is to reshape how PIF literature on underrepresented groups is formed and developed fostering inclusivity and avoiding stereotypes or biases to create safer spaces for REMPs. Examples, reflections, and considerations are provided to strengthen the foundational rationale for claims and recommend cultural humility when creating examples to prevent generalizations, biases, or stereotypes. The authors also recommend considering how cross-racial interactions impact minoritized students to reduce harm in publications and how PIF is taught in various settings. This offers a social justice approach to further the conversation regarding PIF in pharmacy education from a racial and ethnic perspective.

## CRediT authorship contribution statement

**Natalie Rosario:** Writing – review & editing, Writing – original draft, Supervision, Conceptualization. **Joshua Wollen:** Writing – review & editing, Writing – original draft, Conceptualization.

## Declaration of competing interest

The authors declare that they have no known competing financial interests or personal relationships that could have appeared to influence the work reported in this paper.
